# Comparative study of the implementation of tin and titanium oxide nanoparticles as electrodes materials in Li-ion batteries

**DOI:** 10.1038/s41598-020-62505-x

**Published:** 2020-03-26

**Authors:** Félix del Prado, Hanne Flåten Andersen, María Taeño, Jan Petter Mæhlen, Julio Ramírez-Castellanos, David Maestre, Smagul Karazhanov, Ana Cremades

**Affiliations:** 1 Departamento de Física de Materiales, Facultad de CC. Físicas, Universidad Complutense de Madrid, 28040 Madrid, Spain; 2 Insitutt for Energiteknikk, NO-2027, Kjeller, Norway; 3 Departamento de Química Inorgánica I, Facultad de CC. Químicas, Universidad Complutense de Madrid, 28040 Madrid, Spain

**Keywords:** Batteries, Nanoparticles

## Abstract

Transition metal oxides potentially present higher specific capacities than the current anodes based on carbon, providing an increasing energy density as compared to commercial Li-ion batteries. However, many parameters could influence the performance of the batteries, which depend on the processing of the electrode materials leading to different surface properties, sizes or crystalline phases. In this work a comparative study of tin and titanium oxide nanoparticles synthesized by different methods, undoped or Li doped, used as single components or in mixed ratio, or alternatively forming a composite with graphene oxide have been tested demonstrating an enhancement in capacity with Li doping and better cyclability for mixed phases and composite anodes.

## Introduction

Nowadays, Li-ion batteries (LIB’s) are being considered as the most efficient energy storage for portable devices^1–9^. However, an increase in energy density is needed to accomplish other applications such as the energy needs of electric vehicles^9,10^. Besides, some other challenges are still to be solved in order to become the dominating technology for high performance portable, wearable electronics and electric automotive industrial applications^11^. The improvement of the battery performance can be achieved by optimizing the cathode, the electrolytes^2,9,12–14^ and/or the anode materials^1,15,16^. Therefore, one of the main technological strategies is to implement nanoscale materials as anodes in order to avoid the disintegration of the carbon based anodes used in commercial batteries after a number of charge and discharge cycles. The use of nanomaterials has been proposed to accommodate the mechanical stress due to the volume expansion and contraction during the Li intercalation process^17–19^, providing concomitant advantages such as a higher surface to volume ratio with more active sites for Li storage which facilitate the reversible insertion of Li ions and increase Li and electron diffusion. Among others, metal oxide nanoparticles have been proposed as anode materials and satisfactory results have been provided for oxides based on Co, Ni, Cu, Mn, Mo or Fe^20^ (Goriparti* et al*.^1^ and references therein) which storage ability is based in a conversion mechanism through reduction/oxidation reactions of the metal oxide together with the composition/decomposition of Li compounds^15^. However, this group of conversion-based anode materials suffered from volume expansion, low conductivity and large potential hysteresis as main drawbacks although some remarkable results have been obtained by combining into a composite metal oxides nanoparticles and graphene sheets^21–23^. On the other hand, the use of TiO_2_ is another relevant option still under development^1,15,24–26^, which storage capacity occurs through an intercalation path^27–31^. Although providing poorer energy density, it presents high reversible capacity and power density and enables safety implementation in high power batteries. SnO_2_, as well as silicon, is also a very promising anode material with high theoretical energy density, enabling energy storage through an alloying/de-alloying mechanism with Li. The main technological issues for this material are related to the large volume expansion upon cycling, which results into substantial capacity loss. Recently, in order to overcome this issue, Kim* et al*. have reported anodes based on SnO_2_, where some factors as the morphology of nanostructures^17^, the porous size^18^ or its combination with reduced-Graphene Oxide^32^ a fundamental role in the improvement of the LIB’s.

Published results on anodes containing metal oxide nanoparticles are abundant but the results are highly dependent on the material synthesis and their final physicochemical properties and moreover, the values of key magnitudes are sometimes quite dispersed for the same material^1,15,24,29,33^. Therefore, the goal of this study is to contribute with the comparison of several strategies applied to SnO_2_ and TiO_2_ as anode materials. In this work, a comparative study of the implementation of SnO_2_ and TiO_2_ nanoparticles in combination with carbon-based conductive matrices is carried out in order to enhance battery performance and tailor the capacity and cyclability of the LIB’s. First, SnO_2_ and TiO_2_ nanoparticles synthesized by different routes will be characterized as electrode material in homogeneous comparable conditions. Another point is the results of different mixed TiO_2_ and SnO_2_ materials, which are scarcely reported^34,35^. The influence on the battery capacity of the size, phase and defect structure of the nanoparticles will be evaluated. Moreover, added to the implementation of nanoscale materials, the use of an extra source of Li incorporated by doping the nanoparticles with Li is also explored. Finally, the synergy between different materials such as SnO_2_ and TiO_2_, and the formation of a composite with graphene oxide are also investigated, thereby leading to variable tailored battery performance.

## Methods

The SnO_2_ and TiO_2_ nanoparticles used in this work have been synthesized by different routes. In some cases, composite materials of SnO_2_ nanoparticles with GO or SnO_2_/TiO_2_ mixtures were also employed to fabricate the electrodes of CR 2032 type coin cells with Li as counter electrode.

SnO_2_:Lix (0 ≤ x ≤ 30 Li cationic %) nanoparticles have been synthesized by a soft chemistry route based on the hydrolysis^36^ of a certain precursor (SnCl_2_ ⋅ 2H_2_O (Aldrich 99.99%) into the minimum amount of deionized water, then 100 mL of deionized water was added and heated at ~100 °C under stirring. Once the precursor is properly dissolved and homogenized, the desired stoichiometric amount of LiOH (Aldrich 99.99%) in the case of the SnO_2_:Lix doped nanoparticles was added and kept at the same conditions for 2 hours until the hydrolysis reaction is finished, and the greyish powders start to precipitate. Then, the product obtained is washed with deionized water and filtered in a Büchner funnel several times until pH = 7 to discard any presence of anions involved during the synthesis. Finally, the powders were dried at 80 °C for the evaporation of the residual water. In order to obtain the rutile SnO_2_ phase a thermal treatment at 350 °C during 48 hours was performed. The nanoparticles prepared by this chemical method are named as h-SnLix in this paper, where “h” is from hydrolysis and “x” the cat. % of Li. The anatase TiO_2_ phase was obtained using a similar procedure, starting from Ti(Bu)_4_. Moreover, the rutile TiO_2_ nanoparticles were obtained after a thermal treatment at 250 °C during 24 hours in the case of the anatase phase and, at 1000 °C during 24 hours in order to obtain undoped TiO_2_ rutile phase. The nomenclature for the TiO_2_ nanoparticles is h-TiO_2_-a/r, where “h” is from hydrolysis and “a/r” means the phase anatase or rutile, respectively.

Powders containing both undoped nanoparticles, obtained from the hydrolysis method, were mixed from the single counter parts in different weight ratios (3:1, 4:1 and 5:1) (h-SnLi0:h-TiLi0-a) and milled at 400 rpm during 60 min and repeated for 5 times in order to reduce the grain size and homogenize the mixture of powders. The nomenclature employed for these samples is h-SnTi(X:1), where X:1 means the SnO_2_ ratio with respect to TiO_2_.

The (npLix) SnO_2_:Lix (0 ≤ x ≤ 30 cationic %) nanoparticles were prepared via a wet chemical route (based on a modified Pechini’s method^37^) in order to obtain nanopowders with a controlled particle size, morphology and homogeneous chemical composition. The synthesis and characterization of these undoped and Li-doped SnO_2_ nanoparticles have been previously reported^38^.

Graphene oxide (GO) nanosheets were prepared based on a modified Hummers’ method^39^, using the initial starting materials: graphite (flakes), NaNO_3_ (Sigma-Aldrich 99.9%), KMnO_4_ (Sigma-Aldrich 99.9%), H_2_SO_4_ (Sigma-Aldrich 99.9%) and H_2_O_2_ (Sigma-Aldrich 99.9%).

The composite was prepared from the GO and npLix nanoparticles with a ratio of 3:1. The desired stoichiometric amount of GO and nanoparticles were added to a volume dissolution of 200 mL of deionized water and 10% (v/v) of 1-butanol, then ultrasonicated for several hours at 50 °C and, then washed and filtered with deionized water in a Buchner. The final product obtained was dried at 45 °C for 2 hours. Depending on the dopant amount of the nanoparticles, the nomenclature for the composites will be GO-npLix (where x is the Li % cat. in the nanoparticles, from 0 to 30%). The different materials synthesized in this work, were studied by X-Ray diffraction (XRD) using a Panalytical X’Pert Pro Alpha1 instrument with a working radiation of Cu (K_*α*_) (*λ*=1.54158 Å). ICP-OES has been used to determine the Li incorporation in SnO_2_ and TiO_2_ grown by hydrolysis or liquid-mix in an SPECTRO ARCOS equipped with an ORCA optical system in Rowland configuration using an Ar plasma generator at 27 MHz in axial configuration.

In order to evaluate the electrochemical performance of the prepared materials, a slurry was prepared with 60% of the active materials, 20% graphite (which is commonly used due to its improvement in the electronic conductivity and generates more space for the expansion of the active materials), 5% carbon black and 15% CMC binder, and spread onto a copper foil by using a doctor-blade technique. The electrodes were dried under vacuum at 120 °C for 12h. Electrochemical tests were performed in an assembled 2032 coin cell in an argon-filled glove box with lithium metal used as a counter electrode, with a polymer separator (Celgard 3401) and 1 M LiPF_6_ in 1:1 EC/DMC electrolyte (LP30, BASF). 10 wt% fluoroethylene carbonate (FEC) was used as electrolyte additive. The cells were cycled by Galvanostatic Cycling with Potential Limitation (GCPL) at room temperature between 1.0 and 0.05 V at 0.25C-rate, using a battery cycler from Arbin Instruments.

## Results and Disscusion

**Figure 1:**
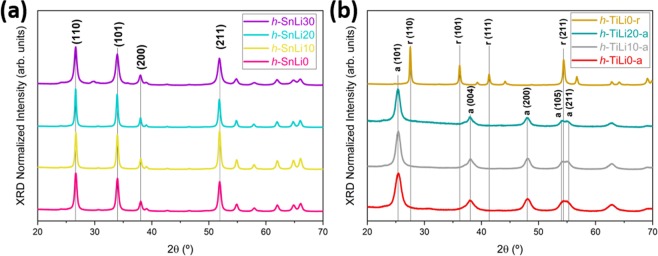
Normalized XRD patterns of (**a**) h-SnLix and (**a**) h-TiLix-(a/r) synthesised by hydrolysis method.

Hydrolysis is a convenient method to synthetize nanoparticles at low temperature and easily scalable. Therefore it has been the first choice to fabricate the SnO_2_ and TiO_2_ nanoparticles used in this work. In both cases, the XRD results are shown in Fig. 1. SnO_2_ nanoparticles doped with Li or undoped as a reference exhibit the rutile structure (Cassiterite, ICDD-04-014-0193) and sizes between 14.9–25.3 nm (see Fig. 1(a)), whereas as grown TiO_2_ nanoparticles crystallize in the anatase phase as shown in Fig. 1(b). The sizes of TiO_2_ anatase (ICDD-00-021-1272) nanoparticles are in the range of 6.2–8.6 nm. If the anatase powder is annealed at 1000 °C during 24 h rutile TiO_2_ (ICDD-00-021-1276) powders are obtained showing a particle size of about 35 nm. The list of estimated crystallite dimensions is included as Supplementary information (Table S1). The SnO_2_ nanoparticles with the highest amount of Li show the lowest dimensions, while for the anatase TiO_2_ the opposite behaviour is observed. No secondary phases or ternary compounds as Li_2_O or Li_2_SnO_3_ were found in the XRD patterns of Li doped nanoparticles as shown in Fig. 1. The residual undefined peak at 30 degrees observed, mainly for h-SnLi30, is due to the romarchite phase of tin oxide. The peaks at 43.5 and 50 degrees are from the sample holder, and the undefined peak around 30 degrees, mainly observed for h-TiLi-a is related to the brookite phase of TiO_2_. All the rest of peaks not explicitly identified are related to the cassiterite SnO_2_, anatase or rutile TiO_2_ as corresponding for each sample.

The amount of Li incorporated in the nanoparticles during the hydrolysis synthesis, has been determined by ICP-OES technique. Table 1 shows the experimental values obtained and the nominal ones are shown for comparison.

**Table 1:** Experimental ICP-OES results obtained for the nanoparticles and its nominal values for comparision.

	h-SnLi0	h-SnLi10	h-SnLi20	h-SnLi30
Nominal (wt. %)	—	0.50	1.08	1.78
Experimental(wt. %)	—	0.017 ± 0.002	0.90 ± 0.03	0.16 ± 0.05
	**h-TiLi0-a**	**h-TiLi10-a**	**h-TiLi20**	**h-TiLi0-r**
Nominal (wt. %)	—	0.92	1.94	—
Experimental(wt. %)	—	0.81 ± 0.05	1.18 ± 0.09	—
	**npLi0**	**npLi10**	**npLi20**	**npLi30**
Nominal (wt. %)	—	0.50	1.08	1.78
Experimental(wt. %)	—	0.45 ± 0.05	0.89 ± 0.09	1.76 ± 0.18

The experimental values obtained from the ICP-OES measurements reveal that the incorporation of Li into the nanoparticles is significantly less than the expected nominal values, and therefore the Li doping is not effectively achieved by the hydrolysis synthesis method. In the case of SnO_2_ the main incorporation of Li is obtained for the 20% cat. doped samples, while in the case of TiO_2_ is the 10% cat. doped samples the one with a higher Li content. As these results are not properly correlated with the nominal ones, they should be taken into account for further discussing the electrochemical behaviour of the corresponding LIB’s.

**Figure 2:**
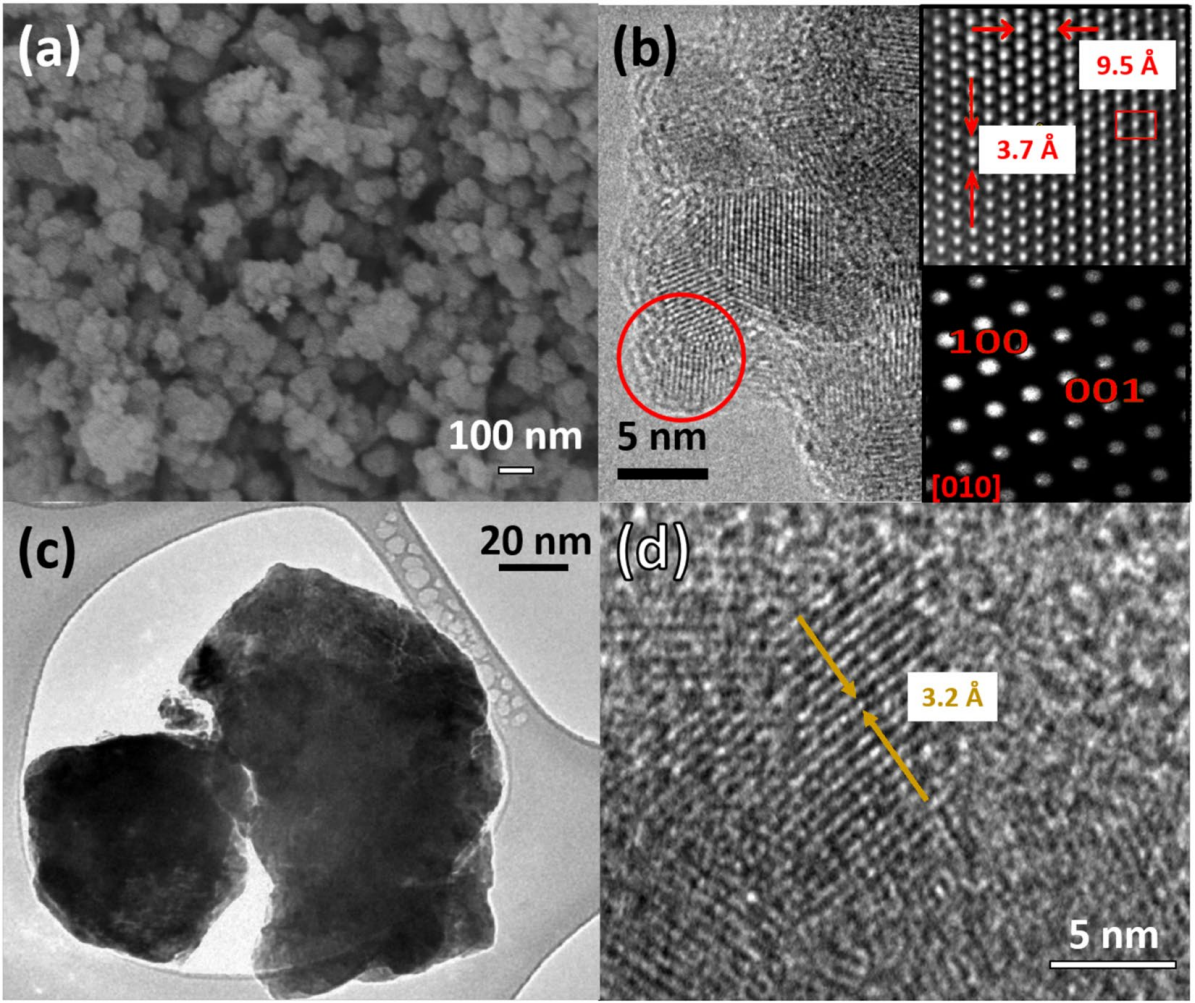
(**a**) SEM and (**b**) TEM images of antatase TiO_2_ samples (h-TiLi0-a), respectively. The upper right inset in (**b**) shows the I-FFT along the axis [010], while the lower right inset shows the SAED pattern of the same sample. In image (**c**) it is shown a TEM image of TiO_2_ rutile (h-TiLi0-r) and in (**d**) HRTEM of the same sample with interplanar distances.

Figure 2(a) shows a large scale of SEM for the samples h-TiLi0-a, where it can be seen conglomerates of nanoparticles forming a mesoporous system. In Fig. 2(b) it can be observed a TEM image of the previous undoped TiO2 anatase sample, showing a small dimensional diameter ~5–7 nm in agreement with the calculated from the XRD technique. The insets in Fig. 2(b) show the interatomic distance of the TiO_2_ anatase crystalline structure (upper right) along the growth direction [010] shown in the SAED pattern (lower right). In the case of the rutile phase h-TiLi0-r, TEM and HRTEM images were acquired in Fig. 2(c,d) respectively. From these images it can be observed that the dimensional size of the nanoparticles is bigger and with an amorphous and undefined shape, while at higher magnification the calculated interplanar distance reveals the rutile phase.

**Figure 3:**
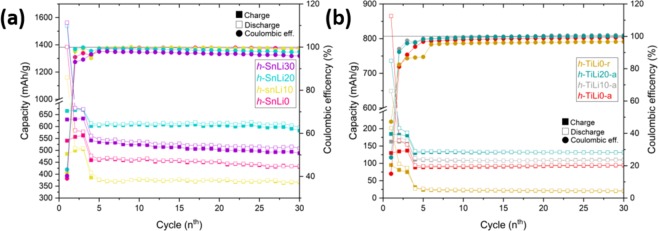
Capacity during the charge/discharge (left axis) and coulombic efficency (right axis) of the nanoparticles (**a**) h-SnLix and (**b**) h-TiLix-(a/r).

The hydrolysis synthesized nanoparticle powders have been used to prepare electrodes of a LIB in a half cell configuration (Table S1 in the Supplementary Information File shows the loading amount on Cu-foil for every sample). The LIB’s were characterized by electrochemical GCPL measurements and the main results of the initial cycles are presented in Fig. 3. (See the Supplementary Information File for curves up to 200 cycles). According to the capacity during the charge/discharge processes shown in Fig. 3, it can be seen that the incorporation of Li in the SnO_2_ and TiO_2_ nanoparticles increases slightly the initial capacity, with the exception of 10% cat. in SnO_2_ due to the low level of Li content effectively achieved in this sample. On one hand, the behaviour in terms of capacity of the h-SnLix cells do not show a linearity (Fig. 3(a)). Initially, for the Li values of 20% and 30% cat. the capacity is larger, ~600 mAh/g and ~500 mAh/g respectively, than for undoped nanoparticles, meanwhile doping with a 10% cat. the capacity it is reduced (~400 mAh/g) even below the value (~463 mAh/g) of the undoped nanoparticles in the cells (h-SnLi0).

On the other hand, when h-TiLix-(a/r) nanoparticles were employed as active material in the electrodes of the LIB’s, it was observed that the capacity increases gradually with the increase of Li incorporated in the case of the anatase phase, while rutile phase shows a very low capacity in comparison with the anatase phase. A possible explanation for this variation on the capacity can be attributed to the different diffusion paths of the Li ions in anatase and rutile crystalline structures. According to previous works^27–30^, the diffusion of Li atoms in TiO_2_-anatase under a potential is best enabled along the lattice directions a and b, while in TiO_2_-rutile the preferential direction is along the c axis, and as a consequence occurs a low volume expansion of ~4%^40^. Further work would be needed to establish diffusion coefficients, for example by employing GITT. Nevertheless, for both set of cells, the best performance was obtained when doping with Li at 20% cat., which seems reasonable since the experimental results obtained from ICP-OES shows that effective amount of Li was higher for these samples. Regarding the capacities reported for SnO_2_ and TiO_2_ materials, our results provide reasonable values over the nominal capacities taking into account the amount of active material in the electrode (see Supplementary Information File), and are quite comparable with the capacities recently reported^24,41,42^. Also the initial capacity values are higher than the theoretical ones specially for the h-SnLi30 and h-SnLi20 and the h-TiLi0-a. Similar behaviour has been observed for mesoporous tin oxide^17,18^.

**Figure 4:**
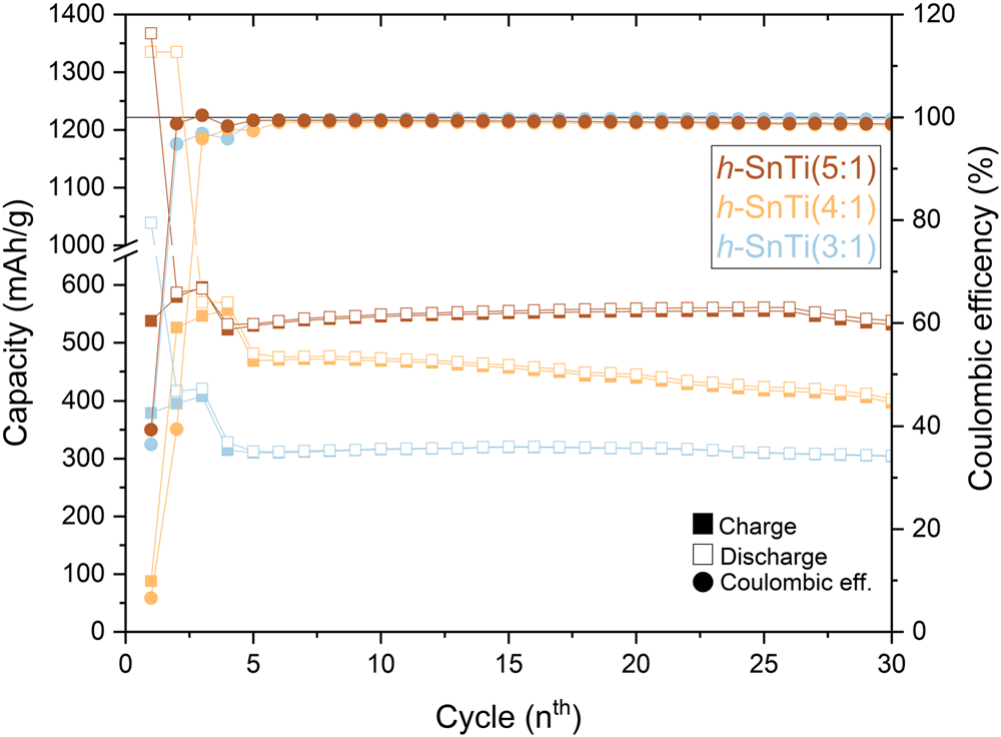
Capacity during the charge/discharge (left axis) and coulombic efficency (right axis) of the mixture h-SnTi(X:1) employed as electrodes in the LIB’s.

Taking advantage of the properties shown by both materials, specifically; the TiO_2_ provides high stability during battery cycling whereas SnO_2_ provides higher capacity, a mixture of h-SnLi0 and h-TiLi0-a nanoparticles in different ratios has been considered in order to improve the previous results as shown in Fig. 4. The capacity in the charge/discharge processes shows a trend according with the ratios used for the active materials in the tested LIB’s, exhibiting a higher capacity when the SnO_2_ to TiO_2_ ratio is increased (X:1). The obtained capacities vary in the initial cycles from ~300 mAh/g (h-SnTi(3:1)) to ~550 mAh/g (h-SnTi(5:1)). The reverse behaviour has been obtained as expected, in terms of stability, where the h-SnTi(3:1) cell shows a lower degradation and better cyclability than the h-SnTi(5:1) cell. Nevertheless, by an easy mixture of powders the behaviour of the LIB’s can be adapted by modifying the ratios to satisfy the desirable energetic response.

The difference on the specific capacities between SnO_2_ and TiO_2_ and the positive effect of Li incorporation, has encouraged us to employ another chemical route, in order to efficiently introduce a higher Li amount in the SnO_2_ nanoparticles.

A Liquid-Mix method^37^ has been employed to produce the nanoparticle powders of SnO_2_ doped with Li with a reduced particle size between 4 and 11 nm. Following this method, a more precise control on the amount of Li incorporated has been achieved, as compared with the hydrolysis synthesized nanoparticles, achieving reasonably the nominal Li values as published elsewhere^38^ and showed in Table I. In this case, the dimensions of the nanoparticles increase as the amount of incorporated Li raises, as a difference with the nanoparticles synthesized by hydrolysis. The incorporation of any dopant into the lattice of a foreign material is usually limited to certain amounts above which segregation of secondary phases or ternary formation take place. The solubility limit of a dopant in a crystalline lattice strongly depends on the experimental growth conditions, which can favor or hinder an effective incorporation. That is the case of the Liquid Mix nanoparticles synthetized by a variation of the Pechini’s method^37^, which favors the dopant incorporation in a very controlled way due to the presence at molecular level of the different metals in the intermediate three-dimensional resin of the precursors in the same stoichiometric relationship that the oxide presents. The final pyrolysis at relatively low temperatures of the obtained product, give rise to the desired composition of the mixed oxide chemically combined in a pure, uniform and finely divided state, explaining the high efficient incorporation of Li in the Liquid Mix samples, which are very close to the nominal amount of Li. A different situation is the case of nanoparticles synthetized by hydrolysis, for which the chemical reactions could include several tin or titanium complexes and compounds with the dopant species in the solution through hydrolysis and condensation reactions, which are very sensitive to the pH conditions, as describe in detail, for example for SnO_2_ nanoparticles^43^. Therefore, part of the dopant concentration would not be effectively incorporated into the obtained nanoparticles.

**Figure 5:**
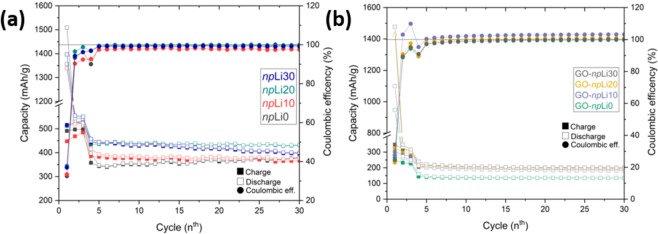
Capacity during the charge/discharge (left axis) and coulombic efficency (right axis) of (**a**) npLix and (**b**) GO-npLix employed as electrodes in the LIB’s.

The results corresponding to npLix electrodes, shown in Fig. 5(a), reveal slight differences in the values of the capacity on the charge and discharge during the first cycles, nevertheless it can be seen that an addition of lithium in the nanoparticles increases gradually the capacity. In the case of the undoped nanoparticles as active material in the electrodes, npLi0, the values of the capacity are ~375 mAh/g, similar to those doped with Li at 10% cat. When the amount of Li present in the nanoparticles is higher, npLi20 and npLi30, the capacity rises until values of ~415 mAh/g. It should be noticed that the measured capacity values are slightly lower when using SnO_2_ nanoparticles synthesized by a liquid-mix method, as compared with those nanoparticles synthesized by hydrolysis. In this case is a general trend that the Liquid-mix method allows to achieve nanoparticles with lower dimensions, which could involve reduced capacity. If capacity values from h-SnLi30 and npLi30, with similar Li concentration, are compared, the higher capacity observed for the former could be related to the larger dimensions and/or the variable concentration of defects (such as oxygen vacancies) in the nanoparticles, promoted by the hydrolysis method. However, cyclability values of the nanoparticles synthesized by Liquid-mix is slightly improved as compared with those from nanoparticles synthesized by hydrolysis (See Fig. S1).

As is well known, SnO_2_ suffers a large volume expansion during the de/lithiation processes^1,9,15,33^ deteriorating the functionality of the electrode and reducing the lifetime of the LIB’s. Other strategy, in order to improve cyclability and life time, is the combination of this material with graphene oxide, as a composite material^21– 23,44^. Fig. 5(b) shows the capacities obtained from those LIB’s, which active material is a composite GO-npLix. As it can be seen, the values of the capacity decrease to the half in comparison with using bare SnO_2_:Lix nanoparticles, being in the case of GO-npLi0 ~ 120 mAh/g, and increasing this value to ~200 mAh/g when the composites contain nanoparticles doped with Li. These low capacity values can be understood based on the lower amount of nanoparticles used in the synthesis of the composite-samples, however the improved performance as a function of the Li concentration is maintained in the composites. Beside this reduction in the capacity, the stability when using a GO host is highly improved toward 200 cycles (See Supplementary Information File) most likely owing to a combination of the well-known mechanical stability of the GO^45,46^ and less strain and SEI cracking during lithiation-delithiation due to a lower capacity per cycle. Applications of pristine GO in LIB’s usually suffer from its low conductivity, which in this case can be improved by adding SnO_2_ nanoparticles while keeping the inherent GO stability.

## Conclusions

In this work, a comparison of the properties of tin and titanium oxides as anode components in Li-ion batteries have been carried out. Different materials and synthesis methods, Li doping and utilization of mixed phases of tin and titanium oxide and composites with graphene oxide have been evaluated and the results related to the size, crystalline phase and surface properties and defects of the materials. In the case of SnO_2_ synthesized by hydrolysis significant capacity ( ~ 615 mAh slightly higher than the nominal capacity for the best cases) and cyclability are achieved by doping nanoparticles with certain amounts of Li, although the amount of Li effectively incorporated in the nanoparticles is far from the nominal concentration. The use of SnO_2_ nanoparticles synthesized by a Liquid-Mix method improves the ciclability but the capacity is slightly lower (~415 mAh), due probably to the reduced size of this nanoparticles as compared to the ones obtained by hydrolysis. On the other hand, the best results for titanium oxide obtained by hydrolysis are observed for the anatase phase but present lower capacity (~125 mAh) values in comparison to the measured values for SnO_2_, although the ciclability is remarkable. By mixing undoped rutile-SnO_2_ and anatase TiO_2_-nanoparticles in different ratios by a mechanical milling a tuneable performance in terms of capacity (between ~300 and ~550 mAh) and cyclability that could be employed in further applications is observed. Finally, by employing composites of GO-SnO_2_:Li a stabilization on the degradation though at the cost of decrease in the capacity values (~200 mAh) is achieved. The different strategies followed in this work enable the obtention Li-ion batteries with SnO_2_ based anodes with good performances. Further tunability could be obtained by altering the ratio of the mixed material and composites.

## Supplementary information

Supplementary Information.
