# Supplementary material for: Comparative study of the implementation of tin and titanium oxide nanoparticles as electrodes materials in Li-ion batteries
Source: Sci Rep. 2020 Mar 26;10:5503. doi: 10.1038/s41598-020-62505-x (PMC7099030; doi:10.1038/s41598-020-62505-x)
Supplement: Supplementary file 1 — Supplementary Information. [file 41598_2020_62505_MOESM1_ESM.pdf]

"Supplementary Information File"

Comparative study of the implementation of tin and titanium oxide nanoparticles as electrodes materials in Li-ion batteries

Félix del Prado[1*], *H. F. Andersen*[2], *M. Taeño*[1], *J.P. Mæhlen*[2], *Julio Ramírez-Castellanos*[3], *D. Maestre*[1], *S. Karazhanov*[2] and *Ana Cremades*[1]

[1] Departamento de Física de Materiales, Facultad de CC. Físicas, Universidad Complutense de Madrid, 28040, Spain.
[2] Insitutt for Energteknikk, NO-2027, Kjeller, Norway
[3] Departamento de Química Inorgánica I, Facultad de CC. Químicas, Universidad Complutense de Madrid, 28040, Madrid, Spain.

*Contacting author´s e-mail: felixdelprado@ucm.es

The synthesized materials employed as active material in the tested electrodes where prepared by different chemical routes. Table S1 shows the average crystallite size estimated by using Scherrer formula from the main diffraction peaks, the % wt. in the electrode, nominal and theoretical capacities for comparison and the amount of load in the Cu-foil in every LIB as well as the capacity due to the main active materials (shorted as a.m.) either SnO_2 or TiO_2 in the electrodes. In the last column of Table S1 it is shown the specific surface area of the active materials obtained by the BET-N_2 technique.

Table S1. Nomenclature employed for the different active materials, averaged particles size, % wt., theoretical, nominal capacities, loading amount of material and capacities due to the compounds (active material = a.m.). Last column shows the specific surface of the nanoparticles obtained by BET.

**Active Material**	**Particle Size (nm)**	**Active Material (% wt.)**	**$C_{theoretical}$ (mAh/g)**	**$C_{nominal}$ (mAh/g)**	**Loading amount on Cu foil (mg)**	**C due to a.m. (mAh/$g_{a.m.}$)**	**C due to graphite (mAh/ $g_{a.m.}$)**	**BET Surface area (m^2/g)**
*np*Li0	4.6	60.1	782	469.7	2.60	346.38	123.22	121.37
*np*Li10	6.0	59.5	782	465.0	3.40	335.53	129.44	61.72
*np*Li20	9.5	60.1	782	469.7	2.56	347.32	122.43	15.32
*np*Li30	10.2	60.1	782	469.7	3.43	346.18	123.65	43.21
h-SnLi0	18.0	59.9	782	468.1	5.82	342.95	125.16	72.80
h-SnLi10	23.0	60.0	782	469.0	4.93	345.19	123.86	15.92
h-SnLi20	25.3	60.0	782	469.1	2.29	345.29	123.83	14.71
h-SnLi30	14.9	60.1	782	469.7	4.09	346.23	123.44	26.28
h-TiLi0-a	6.2	60.0	335	201.0	2.49	76.73	124.27	191.90
h-TiLi10-a	7.9	60.0	335	201.0	2.49	76.88	124.52	141.39
h-TiLi20-a	8.6	60.1	335	201.4	5.39	78.06	123.34	---
h-TiLi0-r	34.7	60.0	335	201.0	2.56	77.48	123.52	2.67
GO-*np*Li0	4.6	58.4	474.5	276.9	5.15	137.31	139.56	---
GO-*np*Li10	6.0	59.9	474.5	284.0	8.77	161.29	122.69	---
GO-*np*Li20	9.5	60.1	474.5	285.1	2.58	161.75	123.38	---
GO-*np*Li30	10.2	59.9	474.5	284.0	1.99	159.52	124.70	---
h-SnTi(3:1)	12.0	60.1	668	401.3	1.81	278.06	123.24	---
h-SnTi(4:1)	25.3	60.0	691	414.7	2.09	290.93	123.77	---
h-SnTi(5:1)	22.9	59.9	705	422.4	2.00	298.67	123.73	---

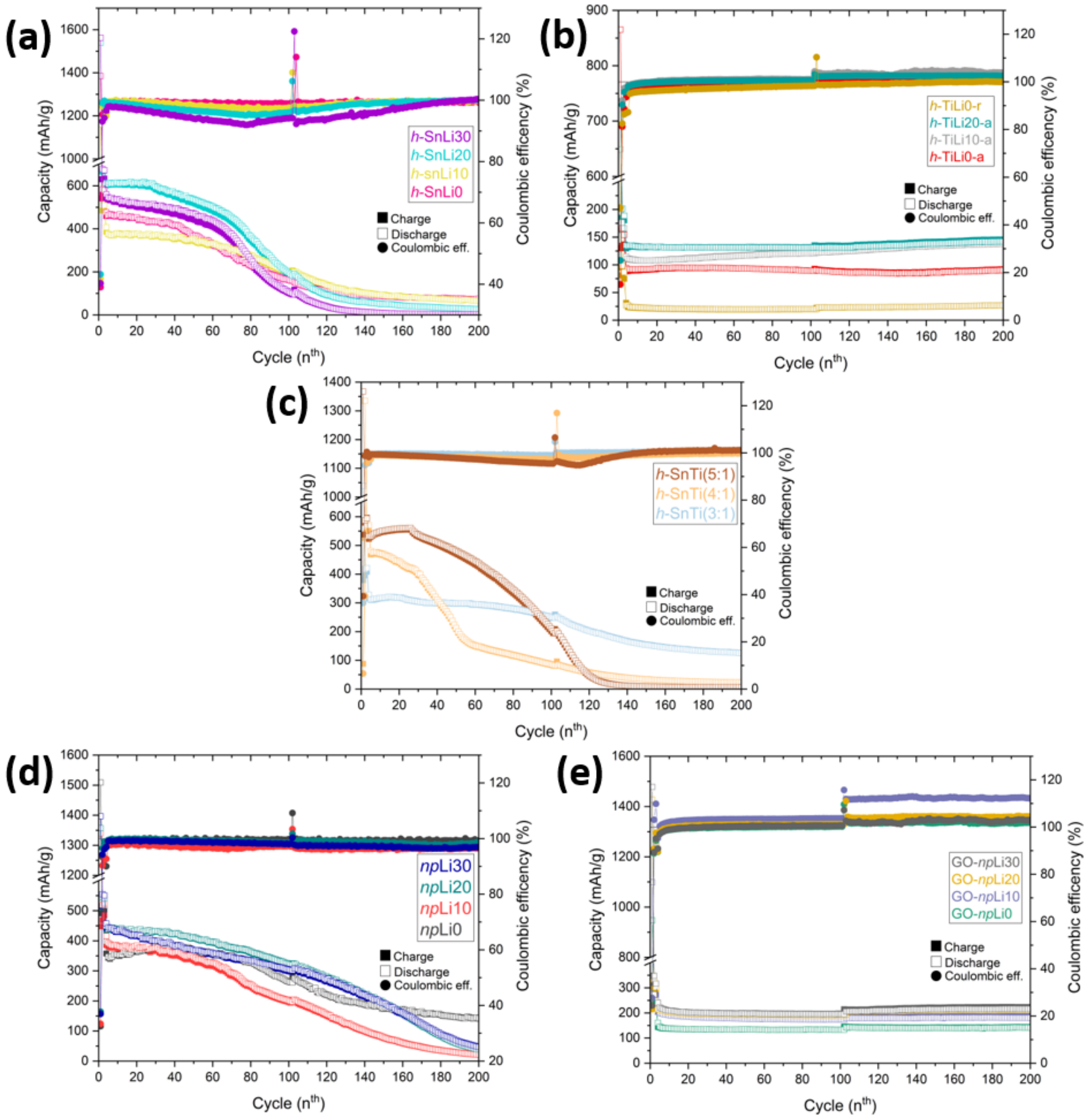

Figure S1. Capacities in the charge (filled squares) and discharge (hollow squares) processes (left axis) and Coulombic efficency (right axis) upon 200 cycles at 0.25C-rate of the cell with active materials: (a) h-SnLix, (b) h-TiLix-(a/r), (c) h-SnTi(X:1), (d) npLix and (e) Go-npLix composites.

The first set of materials that have been studied correspond to SnO_2 nanoparticles without doping and doped with Li, synthesized by hydrolysis method. The curves of charge/discharge and Coulombic efficiency up to 200 cycles can be seen in Figure S1(a). It can be seen that the LIB's that contain nanoparticles with higher content in Li (h-SnLi20 and *h*-SnLi30) are rated slightly higher than expected, 470 mAh/g during the first 70 cycles while the sample *h*-SnLi10 maintains this value slightly below the nominal value.

In the case of the sample without doping, *h*-SnLi0, it remains practically according to the nominal capacity. In addition, it can also be seen that, although for all of them the capacity decreases to 50% approximately between cycles 90 and 100, the LIB's with higher Li content lost practically all its capacity from above cycles, as opposed to those cycled LIB's with lower content in Li, where despite having less capacity, this does not become null unless up to 200 cycle. The Coulombic efficiency that present this set of samples do not differ between them as shown in Figure S1(a), taking values in the first cycle around the 40%. In addition, according to the LIB's with higher Li content, which a-priori are supposed to exhibit improved performance, variations in the Coulombic efficiency below 100% between the first cycles up to approximately the 175th cycle are shown. In contrast to this, the LIB's presenting lower capacity (*h*-SnLi0 and *h*-SnLi10) just presented variations on the Coulombic efficiency, taking values close to 100% from the third cycle to 200 cycles.

The second set of samples which were tested as electrochemical cells, were nanoparticles of TiO_2-anatase without doping and doped with Li, and TiO_2-rutile, synthesized by hydrolysis method. In Figure S1(b) the curves of charge/discharge and Coulombic efficiency of these LIB's in the first 200 cycles are shown. Firstly, the obtained capacity for all LIB's, is less than the rated capacity of ~ 200 mAh/g. Another notable difference that can be seen is that the LIB's containing TiO_2 nanoparticles on rutile phase presented a capacity both in charge and discharge much lower than the LIB's that contain nanoparticles of TiO_2 in phase anatase, being these 2 or 3 times larger. On the other hand, within the LIB's whose electrodes comprise nanoparticles in anatase phase, doping with Li slightly increases its capacity. However, this set of samples, show a great stability in terms of cycling, being it practically constant up to 200 cycles, with the exception of the

h-TiLi10-a sample that shows a slight tendency to increase. This stability is manifested in the Coulombic efficiency, with values very close to 100%, where in the first cycle, there are variations among 15% for specimens in phase rutile and between 25% and 50 % for specimens in phase anatase.

The third set of samples analyzed as LIB's electrochemical cells, were those related to material compounds, formed by a mixture of powders in different ratios, nanoparticles of SnO_2-rutile and TiO_2-anatase, both materials without doping and prepared by hydrolysis method. Their characteristic charge/discharge and Coulombic efficiency curves can be seen in Figure S1(c) up to 200 cycles, where significant differences among them appear. First of all, the capacity for the first ~ 50 cycles is in agreement with the proportions of Sn:Ti present in the active materials used as electrodes, being greater the higher content in Sn there in the Sn:Ti ratio and gradually diminishes. The electrochemical cell corresponding to *h*-SnTi(3:1) presents lower values than the rated capacity of ~ 401 mAh/g, however its stability in terms of cyclability is highest during the 200 cycles, tending to a minimum capacity of ~ 150 mAh/g. In the case of the LIB's named *h*-SnTi(4:1) the nominal capacity is slightly superior to the ~ 415 mAh/g during the first 25th cycles, where then is marked by a accused descent , reaching the 50% in one cycle ~ 50 thereafter the capacity decreases according to the cycles, tending to the total loss or below 100 mAh/g from the cycle number 100th. With respect to the electrochemical cell with highest proportion of Sn:Ti, *h*-SnTi(5:1), it presents a capacity above the corresponding nominal to ~ 423 mAh/g, being these rated values close to the ~ 550 mAh/g in the first 50 cycles, then this capacity begins to slowly decrease until approximately the 100th cycle, where the capacity is reduced to the approximately 50%.

In this cycle appears a spiked point and then the capacity decreases more quickly until the cycle 125th, where it began to be low or negligible up to 200 cycles.

We have studied the next set of electrochemical cells whose electrode contains such active material nanoparticles of SnO_2 without doping and doped with Li, npLix, were synthesized by the method of LM. In Figure S1(d) it can be seen the curves of charge/discharge capacity and the Coulombic efficiency based on cycles, up to 200 cycles. The cells have a nominal capacity both in charging and discharge, usually close to the 470 mAh/g for the first ~ 50 cycles, with the exception of the cell in *np*Li10, which has a capacity slightly lower at all levels compared with other cells. Cells that have a greater capacity to the first 100 cycles are those with higher content in Li, decreasing its capacity to the ~50% in the cycle 150, the cell that maintains a non-null capacity from 200 cycle is the cell of the sample without doping npLi0. With regard to the Coulombic efficiency shown in Figure S1(d), it can be seen that it stays constant and close to 100% from 5th cycle introducing slight variations. From the first cycle this efficiency is between 30% and 40%.

Finally, the set of GO-npLix composites were tested as electrochemical cells LIB's. Then, in Figure S1(e) is shown the corresponding charge/discharge curves, as well as the Coulombic efficiency. These show significant differences. First, all of them are kept below ~ 284 mAh/g (nominal capacity), being the most notable difference for the GO-*np*Li0, that composite containing no Li on *np*Lix nanoparticles, while, even though the difference is lower, a slight increase appears in the capacity with Li content present in nanoparticles *np*Lix. However, all of them present stability up to the 200th cycle. Second, it can be observed a leap and increase in capacity from the 100th cycle, cycle in

which the LIB's rest during 24 hours. This variation is more pronounced in terms of Coulombic efficiency, where in the first cycle presents some variations ranging between 20% and 30% between the different samples. In the case of the sample GO-*np*Li10 occur variations in the 2nd and 3rd cycle above 100% and a more pronounced variation from cycle 100 onwards, reaching values higher than 110%.

In Figure S2 is shown the characteristic voltage drop vs the discharge/charge capacity of the different materials that have been studied as the active material in the cells at a 0.25C-rate.

In the case of SnO_2 nanoparticles appears a plateau between 0.8 up to 1.0 V in the potential (Figure S2(a) and (d)), which classically has been attributed to the conversion reaction involving SnO_2 and Li, and the subsequent formation of Sn-Li_2O nano-compounds. [1-8]:

$$SnO_2 + 4Li^+ + 4e^- \rightarrow 2Li_2O + Sn$$

$$Sn + xLi^+ + xe^- \rightarrow Li_xSn \; (0 \leq x \leq 4.4)$$

The reversible capacity in the lithiation and delithiation of SnO_2 nanoparticles are increased in the first cycles due to the activation process that takes part in the materials due to the aggregation of the SnO_2 nanoparticles. According to some authors[9,10] the activation process involves only a part of the material as in the first lithiation-delithiation process the SnO_2 nanoparticles are crushed into smaller particles driving to a more active material for the next cycles. In our experiments the conversion reaction is not fully complete during the first cycle and about five cycles were needed to achieve the main and more stable values of capacity and Coulombic efficiency here reported. Furthermore, in our results this plateau is larger for the samples named as *np*Lix which contains more amount of Li as a dopant or the particle size increases. However, for the LIB’s samples containing *h*-SnLix nanoparticles prepared by the hydrothermal method, some

discrepancies appear in comparison with the previous one where the width of the plateau at ~1.0 eV does not follow a clear tendency either in the Li content or the particles sizes. On the other hand, for samples based on TiO_2 (h-TiLix-(a/r)) the main reversible reaction can be written as:

$$TiO_2 + xLi^+ + xe^- \rightarrow Li_xTiO_2$$

where a clearly visible plateau on the voltage-capacity curve at 1.8 V vs Li/Li^+ appears for LIB¨s with TiO_2-anatse phase nanoparticles in the first lithiation cycle. Due to that the LIB's were only cycled further between 0.05 and 1.0 V vs Li/Li^+ where only the low voltage tail of this reaction participated, in addition to the reversible lithiation and delithiation of the graphite. Nevertheless, subtracting the contribution from the graphite (~123 mAh/g(TiO_2)) we see a slight increment of the capacity vs. pre-lithiation / Li content. Following the behavior of the cyclability of those LIB's containing a mixture Sn:Ti with different ratios as active material it can be seen from Figure S2(c) the same tendency. The samples with larger Sn:Ti ratio show a plateau at ~1.0 V which is corresponded with SnO_2 whereas in the case of the lower Sn:Ti ratio this plateau vanishes in agreement with the TiO_2 material. In addition, it can be seen a superior capacity when increasing the Sn:Ti ratio. Finally, in Figure S2(e) is shown the LIB's prepared with GO-npLix composites show a plateau at ~0.8 V slightly lower that the nanoparticles from Figure S2(d), even more this plateau does not appear in any of the undoped samples npLi0 and GO-npLi0.

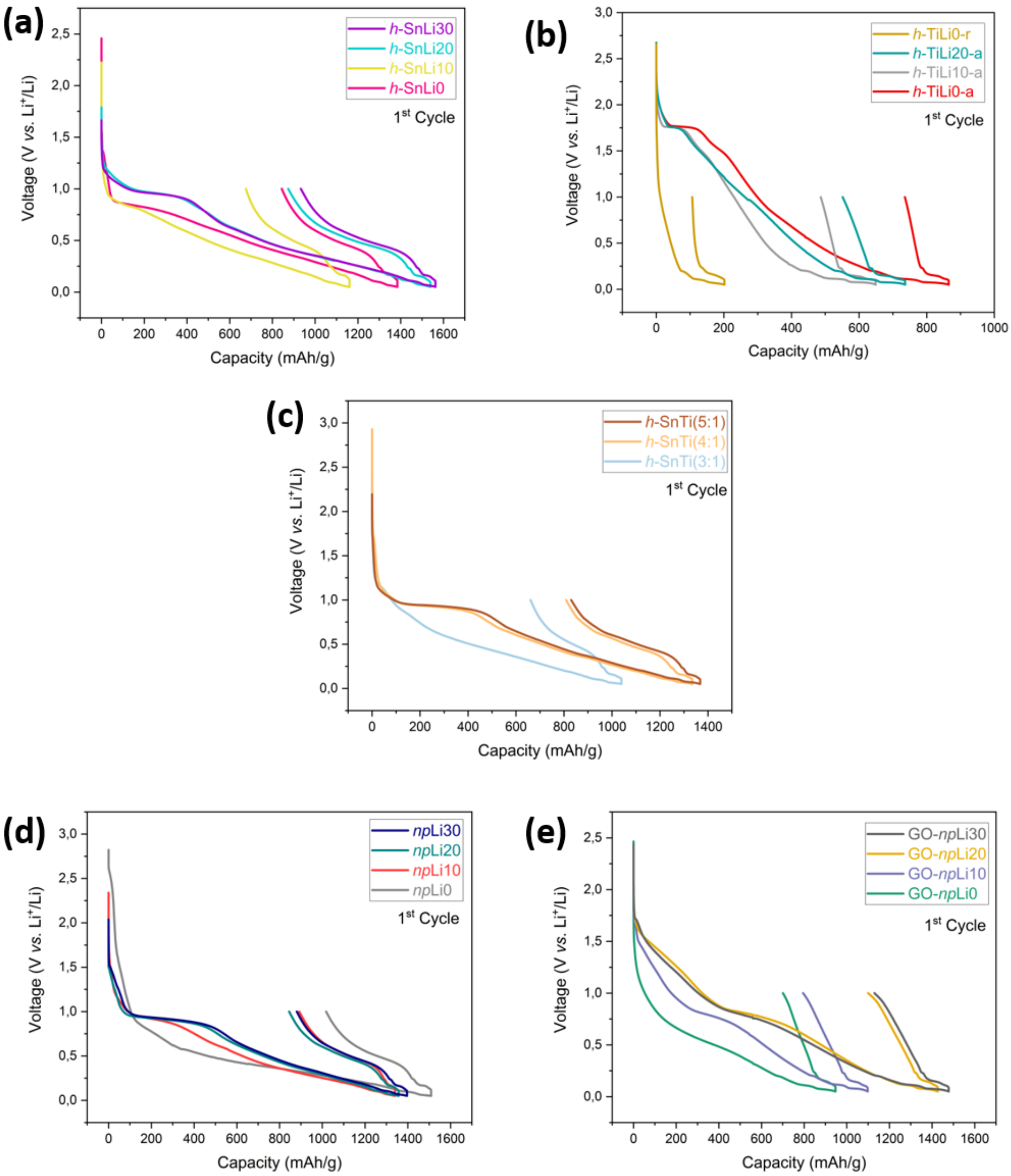

Figure S2. Voltage vs discharge/charge capacity at 0.25C-rate of the cells with active materials: (a) h-SnLix, (b) h-TiLix-(a/r), (c) h-SnTi(X:1), (d) npLix and (e) GO-npLix composites.

References

1 Wang, Y., Lee, J. Y. & Zeng, H. C. Polycrystalline SnO_2 nanotubes prepared via infiltration casting of nanocrystallites and their electrochemical application. *Chemistry of Materials* **17**, 3899-3903, doi:10.1021/cm050724f (2005).

2 Zhang, L.-S. *et al.* Mono dispersed SnO_2 nanoparticles on both sides of single layer graphene sheets as anode materials in Li-ion batteries. *Journal of Materials Chemistry* **20**, 5462-5467 (2010).

3 Chen, Y.-J., Zhu, C.-L., Xue, X.-Y., Shi, X.-L. & Cao, M.-S. High capacity and excellent cycling stability of single-walled carbon nanotube/Sn O 2 core-shell structures as Li-insertion materials. *Applied Physics Letters* **92**, 223101 (2008).

4 Ying, Z., Wan, Q., Cao, H., Song, Z. & Feng, S. Characterization of SnO_2 nanowires as an anode material for Li-ion batteries. *applied Physics letters* **87**, 113108 (2005).

5 Madian, M., Eychmüller, A. & Giebeler, L. Current advances in TiO_2-based nanostructure electrodes for high performance lithium ion batteries. *Batteries* **4**, 7 (2018).

6 Du, Z. F. *et al.* In situ synthesis of SnO_2/graphene nanocomposite and their application as anode material for lithium ion battery. *Materials Letters* **64**, 2076-2079, doi:10.1016/j.matlet.2010.06.039 (2010).

7 Dikin, D. A. *et al.* Preparation and characterization of graphene oxide paper. *Nature* **448**, 457 (2007).
8 Medhekar, N. V., Ramasubramaniam, A., Ruoff, R. S. & Shenoy, V. B. Hydrogen bond networks in graphene oxide composite paper: structure and mechanical properties. *ACS nano* **4**, 2300-2306 (2010).
9 Lian, P. *et al.* High reversible capacity of SnO_2/graphene nanocomposite as an anode material for lithium-ion batteries. *Electrochimica Acta* **56**, 4532-4539, doi:https://doi.org/10.1016/j.electacta.2011.01.126 (2011).
10 Noerochim, L., Wang, J.-Z., Chou, S.-L., Li, H.-J. & Liu, H.-K. SnO2-coated multiwall carbon nanotube composite anode materials for rechargeable lithium-ion batteries. *Electrochimica Acta* **56**, 314-320 (2010).
